# Mohs Micrographic Surgery for Cutaneous Squamous Cell Carcinoma

**DOI:** 10.3390/cancers16132394

**Published:** 2024-06-28

**Authors:** Sven Zürcher, Zora Martignoni, Robert E. Hunger, Michael Benzaquen, S. Morteza Seyed Jafari

**Affiliations:** Department of Dermatology, Inselspital, Bern University Hospital, 3010 Bern, Switzerland

**Keywords:** Mohs surgery, non-melanoma skin cancer, standard excision, squamous cell carcinoma, recurrence

## Abstract

**Simple Summary:**

Cutaneous squamous cell carcinoma is particularly common and its incidence is increasing. Effective treatment is needed to prevent local recurrence and metastasis. The aim of our systematic review was to assess the potential added value of Mohs micrographic surgery compared with conventional excision. The majority of included studies showed a lower risk of recurrence when Mohs micrographic surgery was used to treat cutaneous squamous cell carcinoma. In addition, Mohs micrographic surgery offers advantages for tumors located in aesthetically or anatomically challenging areas. However, this technique requires a certain level of expertise and additional time and resources.

**Abstract:**

Background: The first-line treatment of the localized form of cutaneous squamous cell carcinoma (cSCC) remains surgical excision. Either conventional excision (CE) with margins or Mohs micrographic surgery (MMS) may be preferred, depending on the risk factors of cSCC, the characteristics of the tumor, and the available technical facilities. Methods: This article presents a systematic review of the current literature spanning from 1974 to 2023, comparing outcomes of cSCC treated with MMS versus cSCC treated with conventional excision. Results: Out of the 6821 records identified through the database search, a total of 156 studies were screened, of which 10 were included in the review. The majority of the included studies showed that treatment of cSCC with MMS consistently exhibits a significantly lower risk of recurrence compared to treatment with CE. In addition, MMS is emerging as the preferred technique for the resection of cSCC located in aesthetically or functionally challenging anatomical areas. Conclusion: The studies generally demonstrate that MMS is a safer and more effective treatment of cSCC than CE. Nevertheless, outcomes such as recurrence rates and cost-effectiveness should be assessed more precisely, in order to allow for a more tailored approach in determining the appropriate indication for the use of MMS.

## 1. Introduction

Cutaneous squamous cell carcinoma (cSCC) is notably prevalent and ranks as the second most common type of skin cancer. Its exact incidence is difficult to determine due to the lack of national cancer registries in many countries. However, its incidence is increasing with an aging population, increased sun exposure, and skin cancer screening [1,2]. In the central and southern regions of the United States, deaths attributed to cSCC may be as common as those resulting from oropharyngeal cancer and melanoma [3]. Consequently, this disease poses a significant public health problem. Ultraviolet light (UV) exposure is thought to be a major risk factor for the development of cSCC due to its propensity to cause DNA damage. Other known risk factors include fair skin types, chronic inflammation, immunosuppression, human papillomavirus, age, radiation exposure, smoking, and hereditary disorders [4,5].

Most patients diagnosed with cSCC typically present with localized disease that is amenable to local treatment. However, tumor recurrence, lymph node involvement, or other distant metastases can lead to significant morbidity and mortality. The metastatic rate of cSCC varies across different study series, especially regarding durations of follow-up. A study with more than 6000 patients with a 10-year follow-up found a metastatic rate of 1.9–2.6% [6]. However, Rowe et, al. [7] reported a rate of 5.2% for studies with follow-up durations exceeding 5 years.

Several treatment options have been discussed for localized cSCC. Destructive treatments (curettage and electrodessication, cryotherapy, and photodynamic therapy) are not generally recommended due to their lack of efficacy and associated risk of recurrence. In selected cases, radiotherapy can be a valid alternative to surgery. Surgical treatment remains the gold standard, even in elderly or debilitated patients [8]. Two surgical strategies may be evaluated: conventional excision (CE), which involves excisional margins of 5 to 10 mm and postoperative pathologic evaluation of the margins, or micrographic surgery and its variants. Mohs micrographic surgery (MMS), a well-established surgical technique, offers high cure rates and low risk of recurrence while preserving as much of the surrounding healthy tissue as possible, which is of great importance in cosmetically and anatomically sensitive areas [9]. Horizontal histologic sections are performed, allowing evaluation of the entire lateral and deep margins. After excision, the tumor is immediately oriented, sectioned on a cryostat, fixed, and stained with hematoxylin and eosin. Subsequently, the tissue is microscopically analyzed to evaluate for the presence of cancer cells and the margins are assessed for completeness of excision. If there is a residual tumor, the affected areas are excised. In contrast, with standard excision and “bread loaf” vertical sectioning, only a portion of the margins are histologically evaluated. This article presents a systematic review of the current literature, comparing the treatment outcomes of cSCC using MMS and CE, with a focus on disease recurrence rates.

## 2. Materials and Methods

We performed a systematic review of the literature based on the PubMed database for the relevant published studies using the PRISMA method. We used the search terms “carcinoma, squamous cell”, “SCC”, “mohs surgery”, “MOHS”, and “chemosurgery”, both alone and in combination. We screened papers from 1974, which marks the publication date of Tromovitch’s work on the fresh tissue technique [10], to December 2023. Only papers published in English were included in the review. Publications that did not report a comparative disease recurrence rate between treatments with MMS versus CE were excluded. The selected articles were all peer-reviewed.

We excluded systematic reviews and meta-analyses. Arbitrarily, case series with less than 18 patients (mirroring the number in the Princeps publication by Frederic Mohs) [9] were not included. In addition, articles focusing on genital lesions and ungual tumors were omitted from the analysis due to their specific clinical contexts, with treatment with MMS being less common in Europe.

## 3. Results

**Figure 1:**
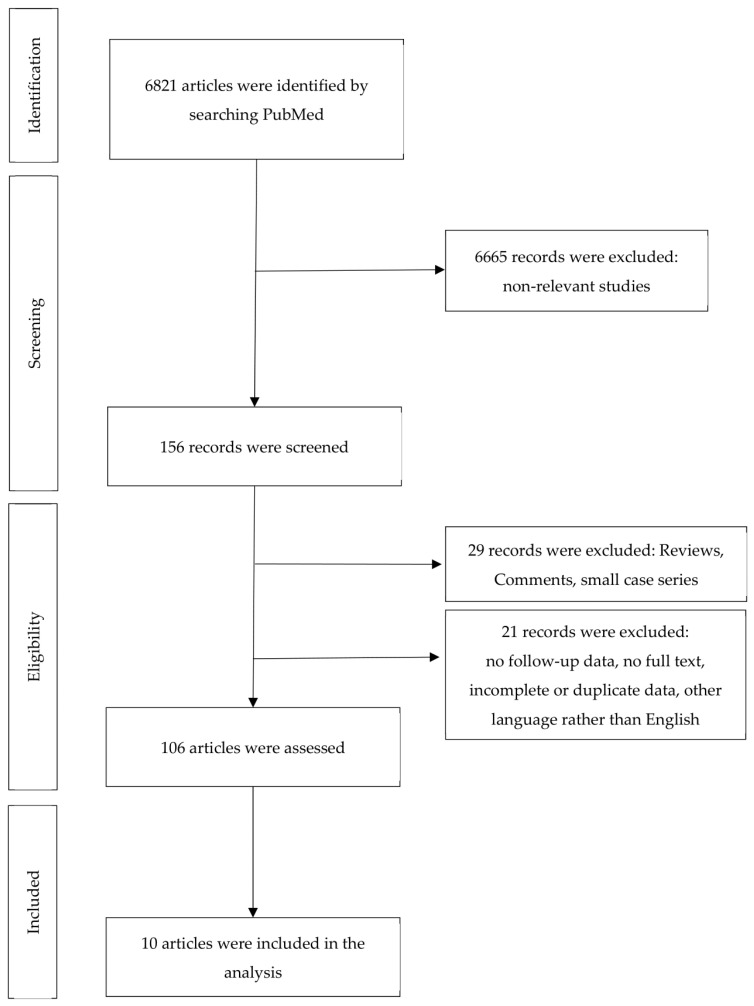
Flow of information during the different phases of systematic review.

A total of 6821 articles were initially identified. Of these, 6665 non-relevant studies were excluded. The full-text evaluation of 156 records yielded 10 eligible articles according to the inclusion and exclusion criteria outlined earlier (Figure 1). The 10 articles cover a total of 6859 tumors treated with MMS and 5048 tumors treated with conventional excisions (in some cases with additional margin control). With the exception of the two studies [11,12] mentioned in Table 1 (use of other techniques), treatment with MMS refers to the “classical” method (bowl-shaped excision, cryostat sectioning), as widely practiced in the USA and Europe and described, for example, in the position paper of the ESMS (European Society for Micrographic Surgery) [13]. According to the established criteria, we did not identify any studies on other variants of micrographic surgery, such as the Munich method (cylindrical excision), the margin strip method (“Tübinger Torte”), and the muffin technique (en bloc excision, separation of margins and base from the unfixed or fixed specimen) [13]. The selected articles are summarized in Table 1 and the main points are discussed there.

### 3.1. Comparison of the Two Methods Regarding Disease Recurrence

Locoregional disease recurrence can contribute to SCC morbidity, especially in cases of advanced local recurrences in anatomically sensitive localizations, particularly the head and neck [15]. Disease recurrence after MMS ranged from 0.7% to 9% in the included studies. However, varying parameters were chosen to assess disease recurrence within the individual study groups. In the study by Sun et al., [18] recurrence rates were similar among the major surgical treatment modalities after excision of eyelid SCC. However, this article compares different methods, including extemporaneous and “slow-Mohs”. Similarly, the excision technique of hand SCC did not substantially influence the outcome of the study by Askari et al. [19] In two other large cohorts of patients with non-melanoma skin cancer (NMSC), the recurrence risk was also not significantly different between MMS and CE. [20,21] However, it is important to note that recurrence rates in these two studies were calculated for all included NMSCs and not specifically for SCC. In van der Eerden’s series, a lag in time was observed between excision and reconstruction for tumors excised by “conventional excision” (CE). Larger tumors benefited from a combination of peripheral and vertical dissection [21]. Thus, a large number of tumors have benefited from the “slow-Mohs” technique. Nevertheless, in other cohort studies, MMS demonstrated a significantly lower likelihood of recurrence than CE [11,12,14,15,16,17]. In addition, MMS exhibited a markedly lower risk of distant metastasis (DM) and disease-specific death (DSD) in a recent large cancer network risk stratification study. [11].

### 3.2. Cost of Treatment

In the study conducted by van Hof et al., [14] results suggest that MMS for the treatment of SCC on the lips is more costly than CE methods, with estimated costs of EUR 3032.24 versus EUR 2564.22, respectively. However, MMS allows excision and reconstruction in one day, resulting in an efficient procedure with a lower risk of recurrence, which should be considered.

### 3.3. MMS for Special Localization and High-Risk SCC

MMS has been shown to be an appropriate method for the surgical removal of cSCC located in cosmetically sensitive or functionally critical areas of the body. This encompasses areas such as the hands and head, with particular emphasis given to delicate areas such as the lips and eyelids. MMS offers a distinct advantage in these locations due to its precision in removing cancerous tissue while preserving healthy surrounding tissue and critical anatomical structures [14,16,18]. This allows for the minimization of resection defects in important areas, such as the nose and eyelid, which, in turn, can facilitate reconstruction [21].

In addition, MMS serves as a valuable treatment option for achieving optimal therapeutic outcomes in high-risk SCC, given that they pose the greatest risk of developing poor outcomes [11]. Interestingly, the study by Stevens et, al. [11] highlights that MMS led to a notable reduction in the risk of adverse outcomes. Furthermore, the experience of Salmon et, al. [12] in the management of sclerosing SCC, a less common variety of neoplasm, suggests that although this subset of carcinoma does not pose a significant metastatic risk, local eradication with conventional surgery may be difficult due to its extensive subclinical extension and propensity for perineural invasion. As a result, they strongly recommended MMS as the primary therapy of choice [12].

## 4. Discussion

Observational studies have consistently demonstrated that treatment with MMS results in a low recurrence rate, [22] especially when compared to the conventional surgical excision of cSCC [11,12,14,15,16,17]. This method is particularly useful in the following cases: as the primary treatment for high-risk cSCC, especially when complete excision is challenging; in cases with an increased risk of recurrence; for tumors with poorly defined borders or aggressive histologic features; and when tissue conservation is essential in aesthetically or functionally sensitive areas [22,23]. Nonetheless, Mohs surgery might not be appropriate for every case, particularly those involving extensive or deeply invasive SCCs, as well as tumors exhibiting satellitosis, a multicentric origin, or skip areas. Managing cases of desmoplastic (or spindle cell subtype) cSCCs remains a challenge. Although one study [12] showed good results with MMS, the use of immunohistochemistry and/or “slow-Mohs” may be a good option. The use of paraffin sections, in addition to fresh tissue analysis, can also be of great value for some difficult cSCC cases [24]. This also highlights the importance of proper patient selection to allow for the most medically effective triage [25].

Although some patients with cutaneous SCC are adequately managed by various modalities, yielding low recurrence rates and minimal potential for poor outcomes, there is a subset of patients with high-risk SCC who face a much greater risk of developing adverse outcomes [11,15]. Previous studies have shown associations between various factors and the rates of local recurrence and metastasis in SCC. These factors include tumor location, size, depth, histologic differentiation, evidence of perineural involvement, recurrence status, precipitating factors other than UV exposure, and host immunosuppression [7,15,18,26]. Patients with high-risk tumors could benefit from MMS, as this method could lead to a lower risk of local recurrence, nodal metastasis, distant metastasis, and disease-specific death in this group [11,15,26]. This is likely because MMS involves histologically reviewing the entire excision margin. In contrast, only a small portion of the excision margin is histologically reviewed after standard excision, increasing the risk of a false negative result (i.e., an undetected incomplete cSCC excision) [16]. It would also be interesting to know more about the benefits of MMS in relation to different risk factors, such as location. Some tumors behave differently depending on their location. More detailed head-to-head studies are needed to answer this question.

Another advantage of MMS over CE, in addition to its excellent tumor clearance and reduced risk of SCC recurrence, is its maximum preservation of healthy tissue [16]. The narrower surgical margins of MMS compared to CE often result in smaller surgical defects [27]. In an interesting study, Bumstead and Ceilley showed that CE removed 180% more tissue than MMS in the treatment of primary skin cancers and 347% more tissue than MMS in the treatment of recurrent tumors [28]. As a result, resection defects in crucial aesthetic and functional areas such as the nose, lips, or eyelids can be minimized, potentially facilitating the reconstruction process [21]. For example, Lee et al. [29] demonstrated the preservation of hand and upper extremity function following MMS with reconstruction for SCC located on the hand or wrist.

Other types of micrographic surgery with paraffin embedding known as “slow Mohs” or “3D histology-guided surgery” are common in Europe. When applying these techniques, histologic results are not available in a single day. However, the method is effective for treating sSCC, resulting in similar results to MMS [30]. Some more detailed literature on this topic is still needed. Comparative studies have been performed on other tumors such as dermatofibrosarcoma protuberens, lentigo maligna, and basal cell carcinoma.

Skin cancer represents a significant health and economic burden to healthcare systems [25,31]. Therefore, it is of increasing interest to determine the most appropriate and cost-effective treatment options for healthcare systems [25]. The controversial differences in the results of these observational studies may be due to methodological shortcomings, varying costs depending on the country of origin of the study, and different pricing methods [22]. This makes it difficult to directly compare the costs of treatment modalities between studies. In general, we have found that the cost of MMS tends to be higher than that of CE, due to the required specialized training and equipment [14]. Cost may also be driven higher if reconstruction is performed by a different specialist, or if multiple Mohs stages per tumor are required for clearance [22]. In the study conducted by van Hof et al., [14] MMS for the treatment of SCC on the lips is more costly than CE methods. However, when considering the increased rates of reoperation due to positive margins, risk of disease recurrence, and functional and cosmetic outcomes, MMS may still be the logical treatment for SCC, especially in cosmetically sensitive areas [14,25]. In line with this, a recent detailed cost-effectiveness analysis of MMS versus CE for intermediate-risk SCC over a 5-year period showed that MMS was less costly and more effective than CE [32].

MMS may be a disadvantage in terms of time, especially in cases requiring multiple stages. The procedure involves a series of steps in which layers of tissue are progressively removed, examined, and mapped to ensure complete removal of the tumor while preserving healthy tissue. However, in most cases, MMS allows for comprehensive tumor treatment and reconstruction within a single day, significantly reducing patient discomfort [21]. Van der Eerden et, al. [21] demonstrated that ninety-four percent of tumors were successfully excised in one or two cycles with MMS, without overburdening the laboratory. All in all, even if individual MMS treatments are more lengthy, excellent work organization and the parallel treatment of several patients should compensate for the additional time requirement.

Another drawback of MMS is that it does not just require the expertise of a specially trained dermatologist but also requires experienced support staff. This may result in limited access to MMS in certain areas due to factors such as geographic location and healthcare infrastructure [33]. The training Mohs surgeons, pathologists, and non-physician personnel demands substantial resources and several months of education and practice to ensure high-quality treatment and diagnosis based on histologic specimens of high technical quality [34].

Several clinics are using “ex vivo confocal microscopy” for surgical margin assessment in order to achieve a faster technique and receive results more quickly [35]. Although this method is already in clinical use and appears promising, its definitive replacement of conventional methods, particularly in cutaneous squamous cell carcinoma, remains to be demonstrated. What is more, the implementation of noninvasive imaging involves additional costs and training.

## 5. Conclusions

In conclusion, despite variations in individual inclusion criteria and study designs across the available research, MMS has many advantages that increase its value. In addition to the advantages of Mohs surgery from a purely oncological point of view, the higher cure rates also provide a sense of security to the patient and physician and avoid the inconvenience and adverse effects of repeated surgical procedures [27]. Moreover, patients treated with MMS tend to report higher long-term satisfaction compared to those receiving other treatments [22]. Compared to other forms of excision, the aforementioned smaller excision defects not only reduce costs but often provide better cosmetic and functional results. This underscores the pivotal role of MMS in achieving optimal outcomes for patients with cSCC, addressing both oncologic concerns and preserving quality of life through meticulous tissue preservation. However, a few points concerning Mohs surgery have yet to be clarified and sometimes give rise to controversy. These points include the necessity for a more accurate assessment of outcomes such as recurrence rates and cost, thereby enabling a more tailored approach to determining the appropriate indication, which may vary depending on the resources. These issues underline the need for future clinical trials with improved methodology, ideally randomized, and sufficient follow-up.

## Figures and Tables

**Table 1:** Summary of included studies.

No.	Study	Type of Study	No. of Tumors	Follow-Up Duration	Disease Recurrence MMS vs. CE	Conclusion
**1**	van Hof et al., 2023 [14]	Retrospective	336 lip SCC 139 treated with MMS, 122 treated with WLE, 75 treated with BT	Median follow-up of 36 months	LR: 2.2% vs. 3.3% RR: 0.7% vs. 6.6%	Considering the cost and risk of locoregional recurrence, MMS would be the most logical treatment for most non-complex T1 lip SCC.
**2**	Stevens et al., 2023 [11]	Retrospective	10,196 SCC 5240 treated with MMS (or PDEMA), 3470 treated with WLE, 1486 treated with other methods	Median follow-up of 27–37 months	MMS or PDEMA had a 35% lower risk of LR, nearly 60% lower risk of DM, and a 45% lower risk of DSD compared with WLE.	MMS (or PDEMA) resulted in lower LR, DM, and DSD compared to WLE. NCCN high and very high-risk groups identify cutaneous SCCs at greatest risk for poor outcomes.
**3**	Xiong et al., 2020 [15]	Retrospective	366 SCC 240 treated with MMS, 126 treated with WLE	Mean oncologic follow-up of 2.8 years	LR: 1.2% vs. 4.0%	MMS provides improved outcomes in T2a SCC. WLE was associated with a 3.3-fold increased risk of local recurrence. Treatment modality, tumor size, and tumor recurrence status are associated with increased local recurrence.
**4**	van Lee et al., 2019 [16]	Retrospective	672 SCC 380 treated with MMS, 292 treated with CE	Median follow-up of 5.7 years	R: 3% vs. 8%	SCC treated with MMS had a three times lower risk of recurrence than those treated with CE (when adjusted for tumor size and deep tumor invasion). MMS may be superior to CE for head and neck cSCC.
**5**	Stuart et al., 2017 [17]	Prospective cohort study	212 SCC 92 treated with MMS; 120 not treated with MMS	Median follow-up of 7.4 years for >90% tumors	Tumor Recurrence: 2.9% vs. 5.5% (adjusted 5-year recurrence rates) *	Recurrence is less common after MMS than after excision, but the absolute difference in recurrence rates is small.
**6**	Sun et al., 2015 [18]	Retrospective	254 cases of eyelid SCC 79 with MMS, 55 with WLE and paraffin section, 49 with WLE and frozen section, 62 with excision alone (without margin control), 9 others	Median follow-up of 40 months	Recurrence rates were similar among the main surgical treatment modalities: WLE and frozen section control, 4.2%; WLE without margin control, 4.6%; MMS, 5.5%; and WLE with paraffin section, 5.5%.	Recurrence rates were similar among the main surgical treatment modalities.
**7**	Askari et al., 2013 [19]	Retrospective	Eighty-six SCC in the wrists, hands, or digits 37 with WLE, 23 MMS, 26 others	Mean follow-up was 6.4 years	N.M.	The technique of tumor excision did not have a major role in outcome.
**8**	Chren et al., 2013 [20]	Prospective cohort study of consecutive patients	1488 NMSC 571 with excision, 556 MMS, 361 others	Median follow-up of 7.4 years	R *^#^*: 2.1% vs. 3.5%	In tumors treated only with excision or MMS, the hazard of recurrence was not significantly different, even after adjustment for propensity for treatment with MMS.
**9**	Salmon et al., 2011 [12]	Retrospective and prospective cohort	73 desmoplastic SCC ** 15 with excision (with or without standard frozen section assessment), 34 MMS (or other micrographic surgery), 7 others	Median follow-up of 3 years	R: 9% vs. 80%	MMS is the surgical modality of choice given the infiltrative nature of cutaneous desmoplastic SCC and the high incidence of perineural invasion.
**10**	van der Eerden et al., 2010 [21]	Retrospective	205 SCC 76 with MMS, 129 with CE (for excised material Smaller < 25 mm, standard random histological examination of deep and lateral margins. For larger diameters, a combination of peripheral and vertical sectioning.)	Median follow-up in the MMS group of 24 months and in the CE group of 16 months	LR + DM: 3.9% vs. 2.3%	MMS and conventional excision are safe in terms of recurrence rates in NMSCs.

BT: Brachytherapy, CE: Conventional excision, cSCC: Cutaneous squamous cell carcinoma, DSD: Disease-specific death, DM: Distant metastasis, LR: Local recurrence, MMS: Mohs micrographic surgery, NM: Not mentioned, NMSC: non-melanoma skin cancers, PDEMA: peripheral and deep en face margin assessment, QoL: Quality of Life, R: Recurrence, RR: Regional recurrence, SCC: Squamous cell carcinoma, SHR: Subhazard ratio, WLE: Wide local excision. * Rates were generally calculated for all tumors included in the study (aggressive BCC, superficial/nodular BCC, invasive SCC, SCC in situ). # Recurrence rates were calculated for all included NMSCs and not specifically for SCC. ** Detailed follow-up information was available for 56 cases.

## Data Availability

The data supporting the results of this study are presented in the current paper.
